# Multifaceted Role of Nef in HIV-Associated Neurocognitive Disorder: Histopathological Alterations and Underlying Mechanisms

**DOI:** 10.3390/brainsci15090987

**Published:** 2025-09-14

**Authors:** Grazia Scuderi, Paolo Fagone, Maria Cristina Petralia, Ferdinando Nicoletti, Maria Sofia Basile

**Affiliations:** 1 Department of Biomedical and Biotechnological Sciences, University of Catania, 95123 Catania, Italy; graziascuderi@hotmail.it (G.S.); paolo.fagone@unict.it (P.F.); 2 Department of Clinical and Experimental Medicine, University of Messina, 98122 Messina, Italy; mariacristina.petralia@unime.it; 3 Department of Medicine and Surgery, “Kore” University of Enna, 94100 Enna, Italy; mariasofia.basile@unikore.it

**Keywords:** Nef, HIV-associated neurocognitive disorder, pathogenetic mechanisms

## Abstract

Although antiretroviral regimens achieve durable suppression of human immunodeficiency virus (HIV) replication, individuals living with HIV remain at an increased risk of developing chronic comorbidities, such as HIV-associated neurocognitive disorder (HAND). In the absence of definitive biomarkers or curative treatments, HAND impacts the survival and quality of life in up to 50% of individuals with HIV. Therefore, novel strategies are highly warranted to improve the diagnosis, monitoring, and treatment of individuals with HAND and a deeper characterization of the still poorly understood pathogenesis of HAND is fundamental to this aim. The pathogenesis, progression, and clinical outcomes of HAND are influenced by different factors, including viral proteins like negative factor (Nef). Among HIV proteins, Nef emerges as a potential key contributor to HAND pathogenesis. Nef could drive specific histopathological alterations in the brain and could be involved in HAND through different interconnected pathogenetic mechanisms. These include: immune dysregulation, oxidative stress, mitochondrial dysfunction, disruption of autophagy, myelin damage and oligodendrocytes dysfunction, blood–brain barrier disruption, alterations of cholesterol homeostasis, and certain potential converging mechanisms with Alzheimer’s disease. Both extracellular and intracellular Nef can contribute to the development of HAND. Interestingly, it has been proposed that Nef may participate in HAND through its incorporation into extracellular vesicles. This review explores the multifaceted role of Nef in HAND, highlighting the histopathological alterations and the pathogenetic mechanisms potentially involved and the potential emerging relevance of Nef as a diagnostic and therapeutic target in HAND.

## 1. HIV-Associated Neurocognitive Disorder (HAND): A Clinical Overview

Human immunodeficiency virus (HIV) infection and its related neurological complications continue to represent a significant global public health issue [1]. As reported by the World Health Organization (WHO), in 2024 an estimated 40.8 million people (between 37.0–45.6 million) were living with HIV worldwide, and around 1.3 million new infections were recorded that year [2]. Even though existing therapies can control HIV infection, individuals living with HIV remain at elevated risk for various chronic comorbidities, including HAND [1]. In the absence of definitive biomarkers or curative interventions, HAND still impacts both the survival and quality of life in up to 50% of individuals with HIV [3]. In particular, a systematic review and meta-analysis evaluating the global burden of HAND in individuals with HIV/acquired immunodeficiency syndrome (AIDS) reported an average prevalence of HAND of 50.41% (95% CI: 45.56, 55.26), with similar rates observed across Europe, Africa, Asia, and the United States [4]. It should be considered that there is a higher risk of HAND with advancing age [5]. Notably, with the increasing accessibility of therapy and the extended life expectancy of patients, the prevalence of HAND is expected to rise, underscoring its growing clinical significance [3].

The term HAND refers to the spectrum of neurocognitive dysfunction associated with HIV infection and encompasses asymptomatic neurocognitive impairment (ANI), mild neurocognitive disorder (MND), and HIV-associated dementia (HAD) [3,5]. Since the introduction of combination antiretroviral therapy (cART), the incidence of HAD, which represents the most severe manifestation of HAND and was previously frequent, has been reduced by 40–50% [5]. However, even in the cART era, HAND remains prevalent, with milder forms predominating, and HAD now rarely observed [5]. In particular, ANI now represents nearly 70% of all HAND forms [5]. The precise causes underlying the persistent prevalence of milder forms of HAND remain unclear [6]. Various hypotheses have been proposed, including incomplete viral suppression within the central nervous system due to limited antiretroviral drug penetration, or the potential neurotoxic effects of the therapy itself [6].

The diagnosis of HAND involves a multimodal assessment combining neuroimaging techniques, cognitive tests, and clinical examinations [7].

The treatment of HAND involves a multidisciplinary strategy designed to target multiple aspects of cognitive dysfunction [7]. The primary therapeutic approach for HAND remains cART, which effectively reduces viral load and prevents further neurocognitive decline [7,8]. Adjunctive treatments, including cognitive rehabilitation, pharmacological support, and psychological interventions, play a pivotal role in treating cognitive symptoms and enhancing patients’ quality of life [7].

The persistence of HAND, despite the widespread use of cART highlights that, while cART has greatly improved the prognosis of individuals with HIV, it is still unable in fully preserving nervous system function [9]. Given that even mild cognitive impairment can have substantial cumulative effects on independence and quality of life, optimizing cART remains a critical priority [9].

Overall, HAND continues to represent a significant unresolved issue for people living with HIV, impacting survival, quality of life, and everyday functioning [5]. Therefore, novel strategies are highly required to improve the diagnosis, monitoring and treatment of individuals with HAND [10].

## 2. Pathogenetic Mechanisms Contributing to HAND

The pathogenetic mechanisms of HAND are still not clear, highlighting the urgent need for further investigation [11]. A deeper characterization of the still poorly understood pathogenesis of HAND is fundamental to identifying effective diagnostic and therapeutic strategies for this significant comorbid condition of HIV infection [12].

**Figure 1:**
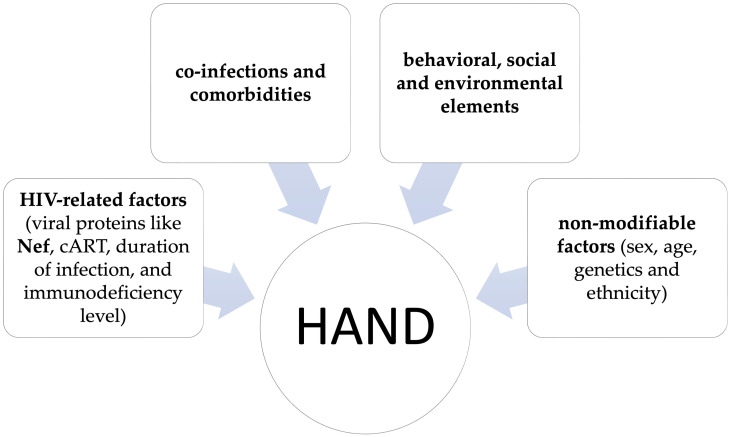
Factors that can affect HIV-associated neurocognitive disorder (HAND) pathogenesis, progression, and clinical outcomes.

It is known that HAND is a complex condition influenced by different factors that can affect its pathogenesis, progression, and clinical outcomes [13]. These factors include: HIV-related factors, including viral proteins like negative factor (Nef), cART, duration of infection, and immunodeficiency level; co-infections and comorbidities; behavioral, social and environmental elements; and non-modifiable factors, such as sex, age, genetics and ethnicity (Figure 1) [13]. Although all these factors significantly impact the disease course, only HIV infection is indispensable for HAND development [13].

In people living with HIV/AIDS low nadir CD4^+^ T cell counts and advanced WHO clinical stages represent potential risk factors for neurocognitive impairment [14]. As CD4^+^ T cells decrease and clinical stage advances, the risk of neurocognitive dysfunction increases [14].

A low level of education, older age, and comorbidity of depression have also been linked with HAND [4]. It is unclear whether the rising incidence of HAND with advanced age could be associated with aging-related immune alterations [15]. In this context, a key issue is whether the primary factor is the immune activation leading to excessive inflammation (inflammaging) or the reduced capacity of the aging immune system to respond to new antigens such as HIV [15].

As regards the pathogenetic role of human host genetics in HAND, it has been shown that different host genes exhibit differential expression throughout the course of HAND [16]. Polymorphisms in various host genes involved in key pathogenic pathways that could be implicated in HAND, including neurotransmitter function, maintenance of nuclear and mitochondrial DNA integrity, telomere length, cytokine and chemokine signaling, and chronic inflammatory responses, have differential effects on the susceptibility and progression of HAND, even though the effect sizes are generally small [16]. Among them, the C-C motif chemokine ligand 2 (CCL2 or monocyte chemoattractant protein 1, MCP-1) gene variant MCP-1-2578G (rs1024611) in HIV-positive individuals, has been associated with faster HIV progression and a 4.5-fold increased risk of developing HAD; however, findings from other studies have been inconsistent, with some reporting no significant impact on neurocognitive function in HAND [16]. Heterozygosity for a variant of C-C motif chemokine receptor 5 (CCR5), the CCR5Δ32 variant, is related to a lower risk of cognitive impairment in individuals with HAND [16]. Certain human leukocyte antigen (HLA) class I polymorphisms have been shown to delay both HIV disease progression and neurocognitive impairment, primarily by encoding CD8 T cell responses targeting more conserved regions of the virus [16]. Some HLA class II variants associated with low CD4 T cell responses to HIV could also be involved [16]. Moreover, the tumor necrosis factor (TNF)-308A host gene variant promotes increased levels of TNF-α and has been implicated in neurotoxicity and progressive neurocognitive impairment in the HIV-infected central nervous system, especially among individuals with HAD [16].

Among the various HIV-related factors, it has been suggested that HIV proteins could play a major role in HAND pathogenesis.

The HIV genome comprises nine genes that encode a total of fifteen viral proteins [1]. Gag, pol, and env give rise to structural proteins (MA, CA, NC), enzymatic components (Pro, RT, IN, RNase H), and envelope proteins (gp120, gp41) [1]. The other genes encode regulatory proteins (Tat and Rev) and accessory proteins (Vif, Vpr, Vpu, and Nef) [1]. Beyond their essential roles in viral replication, these proteins also contribute to the modulation of host cell gene expression, metabolic alteration, and modifications in intracellular signaling pathways [1].

In the initial phases of HIV infection, inflammatory processes disrupt the blood–brain barrier (BBB), enabling the entry of toxic virus, infected immune cells (such as monocytes, macrophages, and T-lymphocytes), and different cellular products from the bloodstream into the brain and, eventually, in the whole central nervous system [17]. Given the absence of resident T-lymphocytes in the brain, HIV can persist for decades within macrophages and astrocytes, forming a stable reservoir of infection [17]. HIV proteins subsequently contribute to neuronal injury through both direct and indirect mechanisms [17]. Neuronal injury in subjects infected with HIV could lead to cognitive, motor, and behavioral impairments, collectively referred to as HAND [18,19].

In particular, HAND is in part mediated by the direct neurotoxic effects of HIV proteins, such as gp120, Tat, Vpr, and Nef, which can be involved in multiple pathways leading to neuronal injury, apoptosis, and dysfunction (reviewed in [20]). These viral proteins exert their effects both directly on neurons and indirectly via interactions with glial cells, promoting neuroinflammation and other harmful processes. For instance, Tat disrupts neuronal calcium homeostasis via NMDA receptor activation, promotes oxidative stress, impairs mitochondrial biogenesis, alters amyloid precursor protein (APP) processing, and stimulates pro-inflammatory signaling in glial cells. gp120 interacts with chemokine receptors on neurons and glia, leading to excitotoxic calcium influx, mitochondrial apoptosis, oxidative damage, and BBB compromise. Vpr contributes by inducing mitochondrial dysfunction, DNA damage, cell-cycle arrest, and chronic neuroinflammation (reviewed in [20]).

Among the HIV proteins, Nef stands out for its potential role in the pathogenesis of HAND, owing to the diverse and multifaceted pathogenic mechanisms it can initiate [13].

Considering the growing evidence of Nef’s involvement in HAND, this review aims to clarify the multifaceted role of Nef in HAND, highlighting the histopathological alterations and the pathogenetic mechanisms potentially involved and the potential emerging relevance of Nef as a promising diagnostic and therapeutic target in HAND.

## 3. Brain Histopathological Changes Induced by Nef

Schenck et al. have recently investigated the specific contributions of the HIV protein Nef to neuroimmune activation, myelin pathology, and neuronal injury using an EcoHIV mouse model, that allows infection of mouse cells [21].

These EcoHIV findings parallel neuropathological and biomarker evidence from people with HAND. Post-mortem studies have consistently documented white matter pallor, microglial nodules, astrogliosis, and neuronal loss in HIV-positive brains [22]. Similarly, biomarkers of monocyte activation and neuroinflammation, frequently elevated in the brains and cerebrospinal fluid of people living with HIV [23,24,25], were also significantly increased in EcoHIV-infected mice. In addition, astrogliosis—commonly observed in HIV-positive brains and associated with astrocytic activation and apoptosis [26]—was recapitulated in EcoHIV models. Finally, non-apoptotic synaptodendritic injury reported in EcoHIV-infected mice [27,28] mirrors hippocampal damage and neurocognitive impairment described in people living with HIV, highlighting the translational significance of these experimental observations.

Histopathological evaluations showed that Nef is a key mediator of neuroinflammatory and degenerative changes in the brain during chronic HIV infection. Compared to EcoHIVΔNef-infected mice, those infected with Nef-competent EcoHIV exhibited pronounced microglial activation, with immunohistochemistry revealing increased IBA-1 (AIF1) expression and hypertrophic microglial morphology within white matter and hippocampal regions. There was a notable upregulation of pro-inflammatory cytokines and chemokines, including interleukin-1β (IL-1β), interleukin-6 (IL-6), CCL2, C-X-C motif chemokine ligand 10 (CXCL10), interferon-γ (IFN-γ), and complement component C3, suggesting that Nef enhances the inflammatory milieu within the central nervous system, which likely contributes to secondary tissue damage [21].

One of the major histopathological findings associated with Nef was white matter damage characterized by myelin impairment. Myelin basic protein immunostaining revealed significant reductions in staining intensity within the corpus callosum of EcoHIV-infected mice, indicating demyelination. Ultrastructural changes, including myelin sheath splitting and disorganization, were consistent with ongoing myelin degradation. In addition, the study reported evidence of oligodendrocyte impairment, with reduced expression of markers associated with oligodendrocyte lineage and function, highlighting a direct or indirect detrimental effect of Nef on cells responsible for maintaining myelin integrity [21].

Astrogliosis, assessed via glial fibrillary acidic protein (GFAP) immunostaining, was present in both Nef-competent and Nef-deficient EcoHIV-infected mice, reflecting a general response to viral infection in the central nervous system. Notably, mice infected with EcoHIVΔNef exhibited even higher GFAP levels compared with both EcoHIV- and mock-infected controls, indicating that EcoHIV can activate astrocytes independently of Nef, likely through other viral proteins such as Tat. At the same time, Nef may exert an inhibitory effect on GFAP expression in white matter, though the underlying mechanisms remain to be elucidated. Given that elevated GFAP is a hallmark of reactive astrogliosis, the reduction observed in Nef-expressing mice suggests a specific modulatory role of Nef on astrocytic function, which may contribute to astrocyte dysfunction during gliotic responses. The differential GFAP expression pattern observed between Nef-competent and Nef-deficient EcoHIV mice raises the possibility that Nef may influence not only astrocytic activation but also the broader dynamics of glial interactions within the central nervous system. Although the available data do not clarify whether this reflects region-specific modulation of astrocytic reactivity or altered astrocyte–microglia crosstalk, both mechanisms warrant future investigation given their potential contribution to HAND pathogenesis.

Neuronal injury was another key histopathological hallmark observed in Nef-expressing EcoHIV-infected mice. Immunohistochemical staining for NeuN demonstrated reduced neuronal marker expression, particularly in the hippocampus, correlating with evidence of neuronal loss and degeneration. Histological analyses revealed morphological signs of neuronal injury, including nuclear condensation and cytoplasmic shrinkage in vulnerable neuronal populations. Additionally, the presence of activated microglia and elevated inflammatory cytokines in proximity to these injured neurons suggests that Nef-induced neuroinflammation may contribute to bystander neuronal damage.

Collectively, these data demonstrate that Nef drives specific histopathological alterations in the brain, including enhanced microgliosis, myelin degradation, oligodendrocyte impairment, and neuronal injury, which are associated with the HAND observed in humans [21]. Indeed, these pathological changes are consistent with Nef’s known cellular functions. Nef has been shown to enhance pro-inflammatory signaling and monocyte/macrophage activation, which could explain the increased microgliosis and elevated IL-1β, IL-6, and CCL2 observed in EcoHIV-infected brains. Its ability to disrupt intracellular trafficking and cytoskeletal dynamics may contribute to oligodendrocyte impairment and myelin disruption, while exosome-associated Nef has been implicated in bystander neuronal injury through the induction of oxidative stress and synaptodendritic damage. Together, these mechanisms provide a plausible molecular basis for the histopathological alterations linked to Nef in the EcoHIV model.

## 4. Potential Pathogenetic Mechanisms of Nef in HAND

Although Nef has traditionally received limited attention in the study of HAND and one study has shown that Nef-deleted HIV-1 is still capable of invading the human central nervous system, emerging evidence indicates that Nef plays a significant role in modulating neuronal function and could be involved in the development of HAND, even in individuals undergoing cART, via multiple complementary mechanisms within the brain [1,29]. Interestingly, Nef seems to be persistently produced and secreted in the blood even in subjects with controlled HIV replication under cART or in elite controllers who naturally control HIV infection, thus representing a potential promising target for HAND [13].

HIV-infected subjects with cognitive impairment exhibit specific structural variants of Nef in comparison with those cognitively stable [1]. The possible identification of a brain-specific Nef structure suggests that genetic variations that modify the protein’s folding or binding properties may contribute to HAD [30]. Nefs in the brain could functionally differ from those in the blood or lymphoid tissues due to adaptation to distinct target cell populations and decreased immune surveillance [31]. Minor adjustments in the physicochemical properties of amino acids across various functional domains contribute to defining a Nef sequence linked to HAND tissues [32].

Nef is a multifunctional polypeptide with a molecular weight of approximately 27–34 kDa [1]. The gene encoding Nef is situated at the 3′ end of the HIV-1 and HIV-2 [1].

Nef was originally thought to function as an inhibitor of viral genome transcription; however, subsequent studies have demonstrated that it is crucial for sustaining high viral loads and contributes to the progression of HIV infection toward AIDS [1]. Nef has been implicated in multiple functions relevant to HIV pathogenesis and it plays a pivotal role in enhancing the pathogenic potential of human immunodeficiency viruses [33,34]. As such, Nef increases the infectivity of HIV particles by approximately tenfold compared to particles produced in its absence, while Nef-defective viruses exhibit a weakened phenotype, characterized by lower viral loads in various experimental models, including murine and monkey models, and in human infections [1,33,35,36,37].

Although it lacks intrinsic enzymatic activity, Nef performs a variety of cellular functions through its interactions with multiple host cell factors [38]. The most extensively characterized functions of Nef stem from its capacity to interact with the cell’s vesicular trafficking machinery and to alter cell signaling pathways [38].

Nef is highly expressed during the early phases of the viral replication cycle and can modulate the surface levels of numerous host proteins, thus disrupting immune responses critical for identifying and controlling viral infection [39]. Nef promotes the downregulation of CD4 by facilitating its uptake in the endosome–lysosome compartment, a function that persists throughout the course of infection and enhances virus infectivity and replication [38]. Moreover, it promotes HIV immune evasion by downregulating the major histocompatibility complex (MHC)-I molecules through a still uncertain mechanism distinct from that involved in CD4 downregulation, thereby protecting infected cells from cytotoxic T lymphocyte killing [38]. In addition, Nef-mediated upregulation of Fas ligand leads to apoptosis in bystander cytotoxic T lymphocytes and both HIV and simian immunodeficiency virus (SIV) Nef also impair MHC class II function [38]. Furthermore, Nef modulates signaling pathways and modifies the activation threshold of lymphocytes by interacting with Src family tyrosine kinases, p21-activated serine/threonine kinases, and Vav [38]. This results in a transcriptional program similar to that induced by T cell receptor stimulation, potentially establishing an intracellular environment that supports viral replication [38].

Multiple studies have demonstrated that exposing neuronal cell cultures to Nef induces neurotoxic effects [30]. Both extracellular and intracellular Nef are thought to play a role in HAND development [12]. Moreover, it has been suggested that Nef may contribute to HAND being incorporated into extracellular vesicles [1]. Nef is readily incorporated into extracellular vesicles and transported by them, allowing the subsequent release of functional Nef protein into neighboring cells, including neurons [1]. It has been suggested that extracellular vesicles released by primary astrocytes can transfer Nef to neurons and play a role in the neurotoxicity linked to HAND development in individuals with HIV infection [40]. A preliminary study assessing if exosomal extracellular vesicles and Nef-containing-exosomal extracellular vesicles detected in plasma and cerebrospinal fluid correlate with the neurocognitive status of aviremic people living with HIV/AIDS has shown that neurocognitive impairment status was associated with the exosomal extracellular vesicles cargo, concentration, and exosomal extracellular vesicles-Nef levels [10].

**Figure 2:**
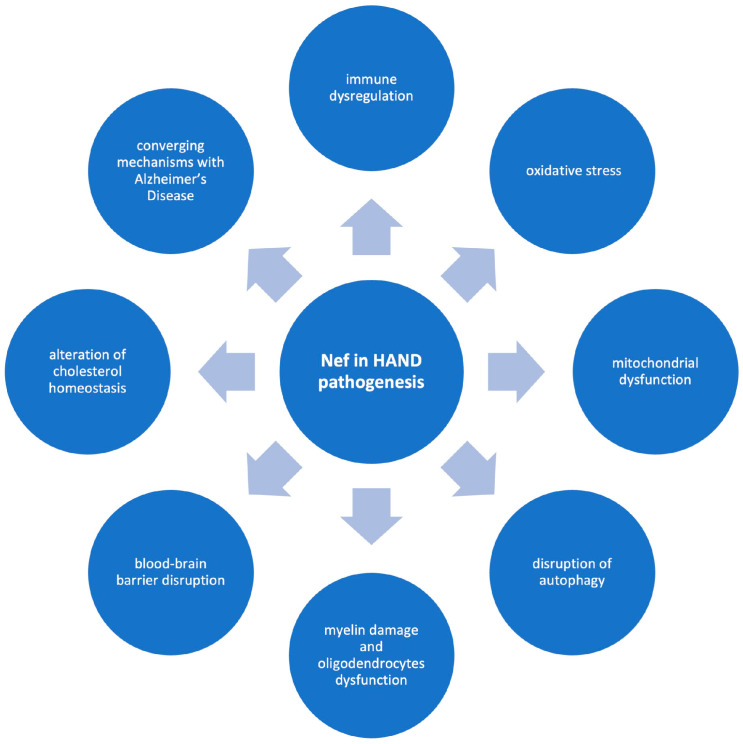
Potential pathogenetic mechanisms of Nef in HAND.

Different mechanisms may underlie the contribution of Nef to the pathogenesis of HAND (Figure 2). These include its effects on immune dysregulation, oxidative stress, mitochondrial dysfunction, disruption of autophagy, myelin damage and oligodendrocytes dysfunction, BBB disruption, alterations of cholesterol homeostasis, and certain potential converging mechanisms with Alzheimer’s disease. The following sections examine each of these mechanisms in greater detail, providing a comprehensive overview of the processes through which Nef contributes to neuropathogenesis. It is important to recognize, however, that many of these mechanisms are not isolated; rather, they are highly interconnected, emphasizing the intricate and multifactorial nature of Nef in the development and progression of HAND. This interdependence highlights the complexity of Nef-mediated neurotoxicity and points to the challenges in delineating precise therapeutic targets within this network of overlapping pathogenic pathways.

### 4.1. Nef and Immune Dysregulation

Numerous studies support the role of Nef-induced immune dysregulation in HAND pathogenesis. One of the main hypotheses to explain HAND pathogenesis attributes it to chronic neuroinflammation linked to systemic low-grade inflammation commonly associated with HIV infection and Nef has emerged as a compelling HIV protein candidate involved in neuroinflammation [21,41]. Nef may act in the brain to induce both local and peripheral inflammatory responses [42]. The interruption of β-adrenergic signaling can decrease peripheral organ inflammation induced by Nef expression in brain astrocytes [42]. Nef has been identified as a key driver of increased neuroinflammation, causing disruption of white matter astrocytes, myelin damage, and synaptodendritic injury in the hippocampus [21].

In particular, one proposed mechanism of HIV-induced neurotoxicity involves the production of pro-inflammatory cytokines by astrocytes/microglia in response to exposure to viral proteins [43]. Interestingly, it has been suggested that pro-inflammatory cytokines could also be involved in the pathogenesis of Neuro-COVID [44].

Nef-expressing astrocytes and activated macrophages can release inflammatory proteins, contributing to neuronal damage and cognitive dysfunction [45]. Furthermore, extracellular Nef released by astrocytes through cell lysis or a regulated process could be involved in neuroinflammation through direct neurotoxic effects or by disrupting the BBB [45]. A disrupted BBB could facilitate an increased influx of inflammatory cytokines from the periphery into the brain [42].

In addition, it has been demonstrated that the intracellular expression of pro-inflammatory cytokines owing to the intracellular expression of Nef virotoxin and the increase in the expression level of kynurenine pathway-specific metabolites could play a key role in Nef-induced neuropathogenesis [46]. The observed reduction in cytokines and kynurenine metabolites following siRNA-Nef interference suggests that RNA interference could be used alongside current cART to help prevent the development of neurotoxicity [46]. Moreover, it has been shown that following the transplantation of Nef-transduced macrophages into the rat hippocampus, Nef promotes monocyte/macrophage recruitment, TNF-α expression, and astrogliosis, and that Nef-induced neurotoxicity correlates with cognitive impairments [47].

It has been found that Nef is involved in neuropathogenesis by directly inducing astrocyte death and indirectly causing neuronal death via the cytotoxic effects of the chemokine interferon γ-inducible protein 10 (IP-10), also known as CXCL10), on neurons [48].

Nef was found to induce thousands of differentially expressed long non-coding RNAs (lncRNAs) in astrocytes [49]. Interestingly, it has been shown that lncRNA AK006025 was implicated in the regulation of the Nef-induced expression of C-X-C motif chemokine ligand 9 (CXCL9), CXCL10, and C-X-C motif chemokine ligand 11 (CXCL11) by interacting with nuclear factor (NF)-κB p65 and CREB-binding protein (CBP)/P300, potentially contributing to neuroinflammation and pathogenesis of HAND [49].

Furthermore, an interesting study has shown that implanting primary astrocytes expressing Nef in the rat hippocampus impaired both spatial and recognition memory and that the memory loss was linked to astrocytic Nef expression, the induction of CCL2, and infiltration of CD163-positive mononuclear cells [45].

It has been suggested that astrocytes expressing Nef induce the expression of transforming growth factor beta-1 (TGFβ-1), which acts in the hippocampus to activate SMAD-2 phosphorylation, leading to the upregulation of CCL2, CD163, and GFAP [50]. CCL2, in turn, recruits perivascular macrophages into the brain, exacerbating inflammation and contributing to neurotoxicity and subsequent learning deficits, thus suggesting that targeting the TGFβ signaling pathway may offer a promising strategy to prevent cognitive impairment in individuals with HAND [50]. Moreover, it has been shown that C-C motif chemokine ligand 5 (CCL5) is markedly induced in SVGA astrocytes transfected with Nef and that the PI3K/Akt and p38 MAPK signaling pathways, together with NF-κB, CEBP, and AP-1, are implicated in Nef-induced CCL5 production in astrocytes [43]. Interestingly, it has also been shown that Nef may alter astrocyte sensitivity to inflammatory molecules via MAPK and JNK pathways, potentially playing a role in the development of neurodegenerative disorders associated with AIDS [17].

It has been suggested that brain-derived Nef sequences undergo adaptive evolution to promote viral replication within brain macrophages and microglia and evade brain-specific immune surveillance [51].

In addition, it has also been suggested that the downregulation of CD4 and MHC-I is likely a key mechanism of Nef within the central nervous system involved in viral replication in the central nervous system and in HAD pathogenesis [52].

Moreover, it has been suggested that Nef may play a critical role in AIDS-related neuropathogenesis mediating the recruitment of leukocytes, which can act both as carriers of the virus and as sources of neurotoxic factors that contribute to disease progression [53].

### 4.2. Nef-Induced Oxidative Stress

It has been suggested that reactive oxygen species (ROS)-induced oxidative stress and cellular damage could represent key factors contributing to HAND development and severity, and that HIV proteins, including Nef, can lead to the production of ROS [54]. In particular, Nef can induce the generation of ROS and trigger accelerated neuronal death, contributing to the onset of HAD [55].

In addition, it has been shown that in microglia, Nef can activate the Vav/Rac/PAK signaling pathway, resulting in the activation of NADPH oxidase 4 (NOX4) and in the production of ROS [54]. The ROS production leads to the accumulation of oxidized molecules such as isoprostanes, aldehydes, and base adducts [54]. This results in disrupted glutamate reuptake in astrocytes, owing to sustained NMDA receptor activation, thereby contributing to indirect neuronal damage [54].

### 4.3. Nef-Associated Mitochondrial Dysfunction

Mitochondrial dysfunction has been proposed as a possible mechanism contributing to HAND in the cART era and Nef has also been associated with mitochondrial dysfunction in the brain [56,57].

It is known that mitochondria are intracellular organelles fundamental for cellular metabolism and play a central role in regulating apoptotic and autophagic signaling pathways [3]. Their importance is particularly pronounced in the brain, where the high energy demands of neuronal activities make mitochondrial function critical for maintaining brain health and cognitive function [3]. In addition, mitochondria are the main source of ROS driving oxidative stress and their dysfunction is considered a fundamental factor in various neurodegenerative diseases [57].

### 4.4. Disruption of Autophagy by Nef

Another proposed mechanism through which Nef could contribute to HAND is autophagy impairment [1]. It is known that autophagy is a dynamic, self-digestive process that captures, isolates, and breaks down intracellular components, including damaged organelles (for example mitochondria), intracellular pathogens, and toxic protein aggregates, to ensure cytoplasmic homeostasis and proper protein quality control [1].

While autophagy can function as an innate defense mechanism, especially in the early stages of viral infection, its dysregulation is often associated with HIV pathogenesis [1].

Chronic exposure to Nef and/or cART may lead to sustained autophagy dysregulation, ultimately causing astrocyte and neuronal dysfunction and playing a role in the pathogenesis of HAND [12]. It has been demonstrated that Nef inhibits the final step of autophagy, thus enabling HIV to evade autophagic degradation in human astrocytes [58].

Nef could inhibit autophagy in various cell types, hindering the degradation of autophagosomes that accumulate impaired organelles and viral proteins, including Nef itself [1]. Since autophagosomes have the ability to merge with multivesicular bodies and release their content through extracellular vesicles, Nef-mediated disruption of autophagy may amplify the effects of extracellular vesicles containing Nef and vice versa [1]. Overall, in addition to its other pathological effects, Nef’s disruption of autophagy and incorporation into extracellular vesicles may facilitate its uptake by neurons, resulting in impaired neuronal function and leading to cognitive decline in individuals with HIV [1].

### 4.5. Nef-Induced Myelin Damage and Oligodendrocytes Dysfunction

It is known that the extent of white matter damage is associated with the severity of neurocognitive impairment in individuals with HAND [59]. It has been demonstrated that Nef-containing extracellular vesicles damage myelin sheaths and impair oligodendrocytes in the murine central nervous system, thus suggesting that Nef extracellular vesicles-induced damage to oligodendrocytes and disruption of myelin integrity may contribute to the pathogenesis of HAND [59].

### 4.6. BBB Disruption Mediated by Nef

Nef seems to cause BBB dysfunction, a major pathological feature of HAND [60,61,62]. In particular, the dysfunction of BBB in the early stages of HIV infection allows viral entry in the brain, infection of brain cells and the ensuing inflammation, oxidative damage of the cells of the central nervous system, neurodegeneration, and the development of HAND [60]. HIV proteins, including Nef, could compromise the integrity of the BBB by disrupting the levels of tight junction proteins, nitric oxide, pro-inflammatory and interferon-inducible genes, leukocyte adhesion, trans-endothelial electrical resistance, and matrix metalloproteinases, ultimately resulting in higher permeability of brain endothelial cells [63].

### 4.7. Nef-Mediated Disruption of Cholesterol Homeostasis

HAND displays the key clinical features of a neurodegenerative disorder, such as progressive neuronal loss, cognitive decline, behavioral changes, and motor deficits [64]. Proposed pathogenic mechanisms for HAND include neuroinflammation, demyelination, apoptosis, and accelerated Alzheimer’s disease development, all of which appear to share disrupted cholesterol metabolism as a common contributing factor [64].

Interestingly, Nef could be involved in HAND pathogenesis by disrupting cholesterol metabolism and altering lipid rafts [13,41,64]. In particular, a critical contribution of Nef extracellular vesicle-induced disruption of cholesterol homeostasis in HAND pathogenesis has been suggested [13].

### 4.8. Converging Pathogenetic Mechanisms Between HAND and Alzheimer’s Disease: A Role for Nef

HAND has several features in common with Alzheimer’s disease, including neuroinflammation, similar transcriptional profiles, and increased levels as well as altered distribution of intracellular beta-amyloid (Aβ), a key element in Alzheimer’s disease pathogenesis [13,41]. Of note, it has also been suggested that the disruption of cholesterol homeostasis and alterations in lipid rafts, which are features of Alzheimer’s disease, along with the involvement of extracellular vesicle–mediated protein transport in both Alzheimer’s disease and HAND, could link the pathogenesis of the two conditions and help explain their shared characteristics [13,63].

Recent evidence suggests that Nef could be involved in shared pathogenetic mechanisms between HAND and Alzheimer’s disease [65]. It has been found that both Nef protein and its mRNA are encapsulated within exosomes that persist in the circulation in individuals with HAD [6]. Nef-containing exosomes derived from the plasma of HAD patients can interact with SH-SY5Y neuroblastoma cells and deliver Nef mRNA [6]. The mRNA could induce Nef expression in target cells, leading to higher expression and secretion of Aβ and Aβ peptides [6]. The augmented secretion of amyloid peptide may be involved in the cognitive impairment observed in HAND [6].

In addition, it has been shown that Nef secreted in extracellular vesicles can be rapidly internalized by neural cells in vitro, decreasing the abundance of the cholesterol transporter ABC transporter A1 (ABCA1) and the cholesterol efflux and raising the abundance and altering lipid rafts in neuronal plasma membranes [41]. Nef secreted in extracellular vesicles induced the redistribution of APP and Tau to lipid rafts and raised their levels, along with those of Aβ_42_, and enhanced tau phosphorylation and the activation of inflammatory pathways [41]. These alterations were associated with impaired neuronal function [41]. Moreover, brief treatment of C57BL/6 mice with purified recombinant Nef or Nef secreted in extracellular vesicles lead to decreased ABCA1 levels and elevated APP abundance in brain tissue [41]. The ABCA1 abundance in brain tissue of HIV-positive individuals with HAND was decreased, and the abundance of lipid rafts was increased in comparison with HIV-negative subjects [41]. Interestingly, APP and Tau levels in brain tissue were found to correlate with the abundance of Nef [41].

Moreover, Nef has been analyzed in postmortem brain samples from subjects with HAND, and it was found to be correlated with the abundance of the lipid rafts marker flotillin 1 and with that of Tau protein phosphorylated on threonine 217 (p-Tau217), an early marker of Alzheimer’s disease, and with HAND severity [65]. Mechanistically, it has been hypothesized a cascade of events that can result in HAND, whereby Nef-containing extracellular vesicles downregulate ABCA1, altering lipid rafts properties and thereby promoting amyloid plaque formation as well as Tau phosphorylation and fibrillization [65].

Overall, these findings highlight potential converging pathogenetic mechanisms between HAND and Alzheimer’s disease, with Nef potentially playing a central role. By disrupting cholesterol homeostasis and lipid rafts and altering amyloid and tau processing, Nef may contribute to HAND pathogenesis through mechanisms similar to those observed in Alzheimer’s disease, thereby extending its possible clinical relevance beyond HAND.

## 5. Conclusions and Future Directions

HAND remains a significant unresolved issue, affecting the survival and quality of life in up to 50% of individuals with HIV [3,5].

Even in the era of cART, HAND is still prevalent, with the predominance of the less severe forms and the rare occurrence of HAD, the most severe form [5].

Currently, there is still a lack of definitive biomarkers and effective specific treatments for HAND, highlighting the need for further research to clarify its pathogenesis and to identify novel diagnostic and therapeutic strategies [5]. The pathogenesis, progression, and clinical outcomes of HAND are influenced by different factors, including viral proteins like Nef [13]. Among the viral factors implicated in HAND, Nef has emerged as a central player since it could to drive histopathological alterations in the brain and could be involved in different interconnected pathogenetic mechanisms in HAND.

These mechanisms encompass immune dysregulation, oxidative stress, mitochondrial dysfunction, impaired autophagy, myelin injury and oligodendrocyte dysfunction, BBB disruption, and altered cholesterol homeostasis [1,17,21,41,42,43,45,46,47,48,49,50,51,52,53,54,55,56,57,59,60,61,62,63,64]. Increasing evidence also supports a role for Nef in mechanisms shared between HAND and Alzheimer’s disease, such as alterations in cholesterol homeostasis, in lipid rafts and in amyloid and tau processing, thereby expanding its potential clinical relevance beyond HAND [6,13,41,65].

Given its diverse pathogenic effects, Nef represents a compelling target for the development of innovative diagnostic and therapeutic strategies. In particular, the molecular characterization of Nef-positive extracellular vesicles in plasma and cerebrospinal fluid may serve as biomarkers for the early detection and monitoring of HAND. The presence of Nef within extracellular vesicles highlights its clinical relevance [6,59,66,67,68], as extracellular vesicles are increasingly recognized as key mediators of intercellular communication in the central nervous system. Importantly, their ability to cross the blood–brain barrier makes them accessible for sampling, offering a unique opportunity to overcome the current lack of reliable laboratory tools to identify patients at risk of neurocognitive decline despite suppressive cART. Such biomarkers could also help stratify patients for early interventions and guide longitudinal monitoring of therapeutic responses.

Beyond biomarker discovery, Nef may also constitute a novel therapeutic target. Although it lacks intrinsic enzymatic activity, its pathogenic effects depend on structural features that enable dimerization, anchoring to cellular membranes, and direct engagement with host signaling partners. These interactions drive a cascade of downstream consequences, including immune dysregulation, impaired autophagy, oxidative stress, and alterations in lipid and cholesterol homeostasis—processes strongly implicated in neuronal injury and cognitive impairment. Pharmacological approaches designed to disrupt these interfaces, such as small molecules or peptides that interfere with Nef dimerization or block its binding to adaptor proteins, are currently in preclinical exploration [69,70,71,72]. In parallel, strategies aimed at neutralizing Nef-containing extracellular vesicles or modulating their uptake by target cells represent an additional therapeutic avenue. While still at an early stage, these efforts raise the prospect of adjunctive interventions that directly mitigate Nef-driven neurotoxicity and complement standard cART.

In conclusion, although further studies are needed to fully delineate the complexity of Nef’s contribution to HAND, current evidence strongly supports its central and multifaceted role in disease pathogenesis. The integration of Nef-focused diagnostics and therapeutics into clinical practice has the potential to transform the management of HAND, shifting from a reactive to a preventive and personalized approach. Overall, the development of integrative, Nef-targeted strategies holds great promise for improving the prevention, diagnosis, and treatment of HAND.

## Data Availability

Not applicable.

## References

[1] Yarandi S.S., Duggan M.R., Sariyer I.K. (2021). Emerging Role of Nef in the Development of HIV Associated Neurological Disorders. J. Neuroimmune Pharmacol..

[2] Sağlık İ., Payaslıoğlu M., Ortaç H., Ayma Rüzgar H. (2025). The Diagnostic Value of Signal-to-Cutoff Ratios in Architect and Alinity HIV Screening Assays: A 10-Year Experience in a Pandemic-Affected, Low-Prevalence Setting. Viruses.

[3] Andhavarapu S., Katuri A., Bryant J., Patel V., Gupta U., Asemu G., Makar T.K. (2020). Intersecting roles of ER stress, mitochondrial dysfunction, autophagy, and calcium homeostasis in HIV-associated neurocognitive disorder. J. Neurovirol..

[4] Zenebe Y., Necho M., Yimam W., Akele B. (2022). Worldwide Occurrence of HIV-Associated Neurocognitive Disorders and Its Associated Factors: A Systematic Review and Meta-Analysis. Front. Psychiatry.

[5] Saylor D., Dickens A.M., Sacktor N., Haughey N., Slusher B., Pletnikov M., Mankowski J.L., Brown A., Volsky D.J., McArthur J.C. (2016). HIV-associated neurocognitive disorder--pathogenesis and prospects for treatment. Nat. Rev. Neurol..

[6] Khan M.B., Lang M.J., Huang M.B., Raymond A., Bond V.C., Shiramizu B., Powell M.D. (2016). Nef exosomes isolated from the plasma of individuals with HIV-associated dementia (HAD) can induce Abeta(1-42) secretion in SH-SY5Y neural cells. J. Neurovirol..

[7] Adhikary K., Banerjee A., Sarkar R., Banerjee R., Chowdhury S.R., Ganguly K., Karak P. (2025). HIV-associated neurocognitive disorders (HAND): Optimal diagnosis, antiviral therapy, pharmacological treatment, management, and future scopes. J. Neurol. Sci..

[8] Elendu C., Aguocha C.M., Okeke C.V., Okoro C.B., Peterson J.C. (2023). HIV-related neurocognitive disorders: Diagnosis, Treatment, and Mental Health Implications: A Review. Medicine.

[9] Sharma I. (2021). Interrogating the impact of combination antiretroviral therapies on HIV-associated neurocognitive disorders. HIV Med..

[10] Caobi A., Werne R., Gomez M., Andre M., Thomas C., Yndart A., Lima-Hernandez F., Nair M., Raymond A.D. (2023). Protein cargo of Nef-containing exosomal extracellular vesicles may predict HIV-associated Neurocognitive Impairment status. Res. Sq..

[11] Petralia M.C., Nicoletti F., Tancheva L., Kalfin R., Fagone P., Mangano K. (2022). Gene Co-Expression Network Modular Analysis Reveals Altered Immune Mechanisms in HIV-HAND. Brain Sci..

[12] Cheney L., Guzik H., Macaluso F.P., Macian F., Cuervo A.M., Berman J.W. (2020). HIV Nef and Antiretroviral Therapy Have an Inhibitory Effect on Autophagy in Human Astrocytes that May Contribute to HIV-Associated Neurocognitive Disorders. Cells.

[13] Sviridov D., Bukrinsky M. (2023). Neuro-HIV-New insights into pathogenesis and emerging therapeutic targets. FASEB J..

[14] Lu Q., Ye Q., Chen D., Li X. (2024). Neurocognitive function and its influencing factors in people living with HIV/AIDS. Zhong Nan Da Xue Xue Bao Yi Xue Ban.

[15] Fulop T., Witkowski J.M., Larbi A., Khalil A., Herbein G., Frost E.H. (2019). Does HIV infection contribute to increased beta-amyloid synthesis and plaque formation leading to neurodegeneration and Alzheimer’s disease?. J. Neurovirol..

[16] Olivier I.S., Cacabelos R., Naidoo V. (2018). Risk Factors and Pathogenesis of HIV-Associated Neurocognitive Disorder: The Role of Host Genetics. Int. J. Mol. Sci..

[17] Saro A., Gao Z., Kambey P.A., Pielnaa P., Marcellin D.F.H., Luo A., Zheng R., Huang Z., Liao L., Zhao M. (2022). HIV-Proteins-Associated CNS Neurotoxicity, Their Mediators, and Alternative Treatments. Cell Mol. Neurobiol..

[18] Puzar Dominkus P., Ferdin J., Plemenitas A., Peterlin B.M., Lenassi M. (2017). Nef is secreted in exosomes from Nef.GFP-expressing and HIV-1-infected human astrocytes. J. Neurovirol..

[19] Ru W., Tang S.J. (2017). HIV-associated synaptic degeneration. Mol. Brain.

[20] Jia F.F., Brew B.J. (2025). Neuropathogenesis of acute HIV: Mechanisms, biomarkers, and therapeutic approaches. Curr. Opin. HIV AIDS.

[21] Schenck J.K., Clarkson-Paredes C., Pushkarsky T., Wang Y., Miller R.H., Bukrinsky M.I. (2025). Nef mediates neuroimmune response, myelin impairment, and neuronal injury in EcoHIV-infected mice. Life Sci. Alliance.

[22] Navia B.A., Cho E.S., Petito C.K., Price R.W. (1986). The AIDS dementia complex: II. Neuropathology. Ann. Neurol..

[23] Ginsberg S.D., Alldred M.J., Gunnam S.M., Schiroli C., Lee S.H., Morgello S., Fischer T. (2018). Expression profiling suggests microglial impairment in human immunodeficiency virus neuropathogenesis. Ann. Neurol..

[24] Guha D., Misra V., Yin J., Gabuzda D. (2023). CSF Inflammation Markers Associated with Asymptomatic Viral Escape in Cerebrospinal Fluid of HIV-Positive Individuals on Antiretroviral Therapy. Viruses.

[25] Kamat A., Lyons J.L., Misra V., Uno H., Morgello S., Singer E.J., Gabuzda D. (2012). Monocyte activation markers in cerebrospinal fluid associated with impaired neurocognitive testing in advanced HIV infection. J. Acquir. Immune Defic. Syndr..

[26] Sabri F., Titanji K., De Milito A., Chiodi F. (2003). Astrocyte activation and apoptosis: Their roles in the neuropathology of HIV infection. Brain Pathol..

[27] Kim B.H., Kelschenbach J., Borjabad A., Hadas E., He H., Potash M.J., Nedelcovych M.T., Rais R., Haughey N.J., McArthur J.C. (2019). Intranasal insulin therapy reverses hippocampal dendritic injury and cognitive impairment in a model of HIV-associated neurocognitive disorders in EcoHIV-infected mice. AIDS.

[28] Kelschenbach J., He H., Kim B.H., Borjabad A., Gu C.J., Chao W., Do M., Sharer L.R., Zhang H., Arancio O. (2019). Efficient Expression of HIV in Immunocompetent Mouse Brain Reveals a Novel Nonneurotoxic Viral Function in Hippocampal Synaptodendritic Injury and Memory Impairment. mBio.

[29] Churchill M.J., Figueiredo A., Cowley D., Gray L., Purcell D.F., Sullivan J.S., McPhee D.A., Wesselingh S.L., Brew B.J., Gorry P.R. (2006). Transcriptional activity of blood-and cerebrospinal fluid-derived nef/long-terminal repeat sequences isolated from a slow progressor infected with nef-deleted human immunodeficiency virus type 1 (HIV-1) who developed HIV-associated dementia. J. Neurovirol..

[30] Lamers S.L., Poon A.F., McGrath M.S. (2011). HIV-1 nef protein structures associated with brain infection and dementia pathogenesis. PLoS ONE.

[31] Agopian K., Wei B.L., Garcia J.V., Gabuzda D. (2007). CD4 and MHC-I downregulation are conserved in primary HIV-1 Nef alleles from brain and lymphoid tissues, but Pak2 activation is highly variable. Virology.

[32] Lamers S.L., Fogel G.B., Liu E.S., Barbier A.E., Rodriguez C.W., Singer E.J., Nolan D.J., Rose R., McGrath M.S. (2018). Brain-specific HIV Nef identified in multiple patients with neurological disease. J. Neurovirol..

[33] Simmons A., Aluvihare V., McMichael A. (2001). Nef triggers a transcriptional program in T cells imitating single-signal T cell activation and inducing HIV virulence mediators. Immunity.

[34] Kandel S.R., Luo X., He J.J. (2022). Nef inhibits HIV transcription and gene expression in astrocytes and HIV transmission from astrocytes to CD4(+) T cells. J. Neurovirol..

[35] Deacon N.J., Tsykin A., Solomon A., Smith K., Ludford-Menting M., Hooker D.J., McPhee D.A., Greenway A.L., Ellett A., Chatfield C. (1995). Genomic structure of an attenuated quasi species of HIV-1 from a blood transfusion donor and recipients. Science.

[36] Gulizia R.J., Collman R.G., Levy J.A., Trono D., Mosier D.E. (1997). Deletion of nef slows but does not prevent CD4-positive T-cell depletion in human immunodeficiency virus type 1-infected human-PBL-SCID mice. J. Virol..

[37] Kestler H.W., Ringler D.J., Mori K., Panicali D.L., Sehgal P.K., Daniel M.D., Desrosiers R.C. (1991). Importance of the nef gene for maintenance of high virus loads and for development of AIDS. Cell.

[38] Basmaciogullari S., Pizzato M. (2014). The activity of Nef on HIV-1 infectivity. Front. Microbiol..

[39] Kwon Y., Kaake R.M., Echeverria I., Suarez M., Karimian Shamsabadi M., Stoneham C., Ramirez P.W., Kress J., Singh R., Sali A. (2020). Structural basis of CD4 downregulation by HIV-1 Nef. Nat. Struct. Mol. Biol..

[40] Sami Saribas A., Cicalese S., Ahooyi T.M., Khalili K., Amini S., Sariyer I.K. (2017). HIV-1 Nef is released in extracellular vesicles derived from astrocytes: Evidence for Nef-mediated neurotoxicity. Cell Death Dis..

[41] Ditiatkovski M., Mukhamedova N., Dragoljevic D., Hoang A., Low H., Pushkarsky T., Fu Y., Carmichael I., Hill A.F., Murphy A.J. (2020). Modification of lipid rafts by extracellular vesicles carrying HIV-1 protein Nef induces redistribution of amyloid precursor protein and Tau, causing neuronal dysfunction. J. Biol. Chem..

[42] Rivera-Ortiz J., Pla-Tenorio J., Cruz M.L., Colon K., Perez-Morales J., Rodriguez J.A., Martinez-Sicari J., Noel R.J. (2021). Blockade of beta adrenergic receptors protects the blood brain barrier and reduces systemic pathology caused by HIV-1 Nef protein. PLoS ONE.

[43] Liu X., Shah A., Gangwani M.R., Silverstein P.S., Fu M., Kumar A. (2014). HIV-1 Nef induces CCL5 production in astrocytes through p38-MAPK and PI3K/Akt pathway and utilizes NF-kB, CEBP and AP-1 transcription factors. Sci. Rep..

[44] Tancheva L., Petralia M.C., Miteva S., Dragomanova S., Solak A., Kalfin R., Lazarova M., Yarkov D., Ciurleo R., Cavalli E. (2020). Emerging Neurological and Psychobiological Aspects of COVID-19 Infection. Brain Sci..

[45] Chompre G., Cruz E., Maldonado L., Rivera-Amill V., Porter J.T., Noel R.J. (2013). Astrocytic expression of HIV-1 Nef impairs spatial and recognition memory. Neurobiol. Dis..

[46] Jadhav S., Makar P., Nema V. (2022). The NeuroinflammatoryPotential of HIV-1 NefVariants in Modulating the Gene Expression Profile of Astrocytes. Cells.

[47] Mordelet E., Kissa K., Cressant A., Gray F., Ozden S., Vidal C., Charneau P., Granon S. (2004). Histopathological and cognitive defects induced by Nef in the brain. FASEB J..

[48] van Marle G., Henry S., Todoruk T., Sullivan A., Silva C., Rourke S.B., Holden J., McArthur J.C., Gill M.J., Power C. (2004). Human immunodeficiency virus type 1 Nef protein mediates neural cell death: A neurotoxic role for IP-10. Virology.

[49] Zhou F., Liu X., Zuo D., Xue M., Gao L., Yang Y., Wang J., Niu L., Cao Q., Li X. (2018). HIV-1 Nef-induced lncRNA AK006025 regulates CXCL9/10/11 cluster gene expression in astrocytes through interaction with CBP/P300. J. Neuroinflamm..

[50] Chompre G., Martinez-Orengo N., Cruz M., Porter J.T., Noel R.J. (2019). TGFbetaRI antagonist inhibits HIV-1 Nef-induced CC chemokine family ligand 2 (CCL2) in the brain and prevents spatial learning impairment. J. Neuroinflamm..

[51] Olivieri K.C., Agopian K.A., Mukerji J., Gabuzda D. (2010). Evidence for adaptive evolution at the divergence between lymphoid and brain HIV-1 nef genes. AIDS Res. Hum. Retroviruses.

[52] Gray L.R., Gabuzda D., Cowley D., Ellett A., Chiavaroli L., Wesselingh S.L., Churchill M.J., Gorry P.R. (2011). CD4 and MHC class 1 down-modulation activities of nef alleles from brain- and lymphoid tissue-derived primary HIV-1 isolates. J. Neurovirol..

[53] Koedel U., Kohleisen B., Sporer B., Lahrtz F., Ovod V., Fontana A., Erfle V., Pfister H.W. (1999). HIV type 1 Nef protein is a viral factor for leukocyte recruitment into the central nervous system. J. Immunol..

[54] Buckley S., Byrnes S., Cochrane C., Roche M., Estes J.D., Selemidis S., Angelovich T.A., Churchill M.J. (2021). The role of oxidative stress in HIV-associated neurocognitive disorders. Brain Behav. Immun. Health.

[55] Jadhav S., Nema V. (2021). HIV-Associated Neurotoxicity: The Interplay of Host and Viral Proteins. Mediat. Inflamm..

[56] Fields J.A., Ellis R.J. (2019). HIV in the cART era and the mitochondrial: Immune interface in the CNS. International Review of Neurobiology.

[57] Villeneuve L.M., Purnell P.R., Stauch K.L., Callen S.E., Buch S.J., Fox H.S. (2016). HIV-1 transgenic rats display mitochondrial abnormalities consistent with abnormal energy generation and distribution. J. Neurovirol..

[58] Saribas A.S., Khalili K., Sariyer I.K. (2015). Dysregulation of autophagy by HIV-1 Nef in human astrocytes. Cell Cycle.

[59] Schenck J.K., Karl M.T., Clarkson-Paredes C., Bastin A., Pushkarsky T., Brichacek B., Miller R.H., Bukrinsky M.I. (2024). Extracellular vesicles produced by HIV-1 Nef-expressing cells induce myelin impairment and oligodendrocyte damage in the mouse central nervous system. J. Neuroinflamm..

[60] Annadurai N., Kanmogne G.D. (2024). Structural and Functional Dysregulation of the Brain Endothelium in HIV Infection and Substance Abuse. Cells.

[61] Atluri V.S., Hidalgo M., Samikkannu T., Kurapati K.R., Jayant R.D., Sagar V., Nair M.P. (2015). Effect of human immunodeficiency virus on blood-brain barrier integrity and function: An update. Front. Cell Neurosci..

[62] Bergonzini V., Calistri A., Salata C., Del Vecchio C., Sartori E., Parolin C., Palu G. (2009). Nef and cell signaling transduction: A possible involvement in the pathogenesis of human immunodeficiency virus-associated dementia. J. Neurovirol..

[63] Kodidela S., Gerth K., Haque S., Gong Y., Ismael S., Singh A., Tauheed I., Kumar S. (2019). Extracellular Vesicles: A Possible Link between HIV and Alzheimer’s Disease-Like Pathology in HIV Subjects?. Cells.

[64] Sviridov D., Mukhamedova N., Makarov A.A., Adzhubei A., Bukrinsky M. (2020). Comorbidities of HIV infection: Role of Nef-induced impairment of cholesterol metabolism and lipid raft functionality. AIDS.

[65] Pushkarsky T., Ward A., Ivanov A., Lin X., Sviridov D., Nekhai S., Bukrinsky M.I. (2022). Abundance of Nef and p-Tau217 in Brains of Individuals Diagnosed with HIV-Associated Neurocognitive Disorders Correlate with Disease Severance. Mol. Neurobiol..

[66] Lee J.H., Schierer S., Blume K., Dindorf J., Wittki S., Xiang W., Ostalecki C., Koliha N., Wild S., Schuler G. (2016). HIV-Nef and ADAM17-Containing Plasma Extracellular Vesicles Induce and Correlate with Immune Pathogenesis in Chronic HIV Infection. eBioMedicine.

[67] Vanpouille C., Brichacek B., Pushkarsky T., Dubrovsky L., Fitzgerald W., Mukhamedova N., Garcia-Hernandez S., Matthies D., Popratiloff A., Sviridov D. (2024). HIV-1 Nef is carried on the surface of extracellular vesicles. J. Extracell. Vesicles.

[68] McNamara R.P., Costantini L.M., Myers T.A., Schouest B., Maness N.J., Griffith J.D., Damania B.A., MacLean A.G., Dittmer D.P. (2018). Nef Secretion into Extracellular Vesicles or Exosomes Is Conserved across Human and Simian Immunodeficiency Viruses. mBio.

[69] Emert-Sedlak L.A., Shi H., Tice C.M., Chen L., Alvarado J.J., Shu S.T., Du S., Thomas C.E., Wrobel J.E., Reitz A.B. (2022). Antiretroviral Drug Discovery Targeting the HIV-1 Nef Virulence Factor. Viruses.

[70] Bouchet J., Herate C., Guenzel C.A., Verollet C., Jarviluoma A., Mazzolini J., Rafie S., Chames P., Baty D., Saksela K. (2012). Single-domain antibody-SH3 fusions for efficient neutralization of HIV-1 Nef functions. J. Virol..

[71] Emert-Sedlak L.A., Moukha-Chafiq O., Shi H., Du S., Alvarado J.J., Pathak V., Tanner S.G., Hunter R.N., Nebane M., Chen L. (2022). Inhibitors of HIV-1 Nef-Mediated Activation of the Myeloid Src-Family Kinase Hck Block HIV-1 Replication in Macrophages and Disrupt MHC-I Downregulation. ACS Infect. Dis..

[72] Shi H., Tice C.M., Emert-Sedlak L., Chen L., Li W.F., Carlsen M., Wrobel J.E., Reitz A.B., Smithgall T.E. (2020). Tight-Binding Hydroxypyrazole HIV-1 Nef Inhibitors Suppress Viral Replication in Donor Mononuclear Cells and Reverse Nef-Mediated MHC-I Downregulation. ACS Infect. Dis..

